# Remarks Concerning the Hygiene of Military Hospitals, Extracted from a Paper Read before the Imperial Academy of Medicine at Paris

**Published:** 1864-02

**Authors:** M. H. Baron Larrey, J. L. Cabell

**Affiliations:** Paris, 1861; University of Virginia, Surgeon P. A. C. S.


### CHRONICLE OF MEDICAL SCIENCE.

Art. I.—*Remarks concerning the Hygiene of Military Hospitals, extracted from a paper read before the Imperial Academy of Medicine at Paris, by* M. H. Baron Larrey. Paris, 1862. [Translated from the French and abridged by Professor J. L. Cabell, University of Virginia, Surgeon P. A. C. S.]

In a few prefatory remarks, the author cites the titles of the various English and French works which either expressly or incidentally treat of the hygiene of hospitals, and, alluding to the incontestible superiority of the English over the French civil hospitals, ascribes this superiority to two predominant conditions of salubrity, namely, the smaller size of English hospitals in general, and a proportionate diminution of the number of beds in each ward, with a consequently more expanded distribution of the sick, and a greater amount of cubical feet of air for each patient on the one part, and, on the other hand, a better selected and more varied system of alimentation.

"At the present time there are nearly one hundred military hospitals distributed over France and Algeria. Some of these are called *permanent* hospitals for times of peace—others are termed *temporary*, and are subdivided into hospitals of the first, second and third line, and these, in times of war, ought to be sufficiently near to each other to facilitate their evacuation and prevent the dangers incident to over-crowding. The fatal effects of the absence of such advantages in the casemated hospitals of the closely besieged cities were conspicuously seen on the occasion of the surrender of the citadel of Antwerp."

"All the French military hospitals, except the small establishments of which I do not propose to speak, include severally the various classes of sick and wounded belonging to the army, there being no special hospitals for distinct classes of disease. But, in each hospital, the regulations require that there should be a separation of the *sick* from the *wounded*. Commonly, too, one or two sections are set apart for venereal cases, isolated wards for patients affected with scabies, others for small pox patients, and private rooms for the gravest cases of fever, &c. Such an arrangement seems appropriate and beneficial, as having a tendency to facilitate the execution of the regular duties of hospital service, to favor disciplinary supervision, and to promote the salubrity of the ward ."

In proof and illustration of the proposition, that a hospital, presenting every other condition of salubrity may be exposed to all the dangers of the most malignant hospital epidemics, the author, after referring to the excellent sanitary condition of the military hospital of Val-de-Grace, at Paris, before the occupation of that city by the allies in 1814, states that, on that occasion, this, as well as the other hospitals, were over-crowded, and hospital gangrene prevailed to such an extent, that nearly every case of amputation proved fatal. Referring to another military hospital in Paris (Gras-Caillou), and adverting to the favorable sanitary influences by which it was surrounded and which was exemplified by the rapid cure of the wounded from the three days' fight of July, 1830, he proceeds to say that "these advantages could not, however, over-ride the opposite influences springing from an accumulation of the sick, as has been recently shown when the board of health found it necessary to propose to the minister of war appropriate remedial measures—namely, the discharge of all the convalescent patients, the reduction of the number of beds and the construction of other hospitals," which measures were promptly taken with the most beneficial results.

"Each army corps possesses an infirmary, in which are treated all benignant affections or diseases of so mild a character as not to require admission into a regular hospital. A two-fold advantage results from this place. In the first place, this class of patients are thereby exempted from exposure to the *nosocomial* effluvia of the hospital, and, on the other hand, the hospitals are preserved from a dangerous accumulation of the sick."

[N. B. At the general hospital, Charlottesville, this principle has been and is now carried out by permitting quite a large proportion of convalescing patients to remain *in private quarters*, reporting in person every few days. With proper care on the part of the surgeon in charge, no evil can result, but only unmixed good. If elsewhere the system works badly through a want of proper attention on the part of the surgeon, he should be held responsible, and not the system abandoned. If the permit in each case designates the particular temporary residence of the patient, and its validity be limited to a specified number of days, it will be very easy for the surgeon to report to the provost marshal or enrolling officer, all who fail to report at the proper time. I find the system to work exceedingly well, though, of course, it gives the surgeon some trouble.]

Adverting to the admirable naval hospitals of France, the author proceeds to say—"That for them, as well as for army hospitals, and indeed for all hospitals, the question which overrides all others, and which ought always to engage the earnest attention of the administrative authorities, is that of *overcrowding* the wards. We may, in the most manifest manner, produce argument, diminish or suppress its effects, by increasing or diminishing the number of sick in a given ward. Our learned and modest colleague, M. Rynard, inspector-general of the sanitary service of the navy, assures me that he has many times in his own experience signalized these conclusive results."

"The application of the principal rules of hygiene to military hospitals presents a question of some complexity, which, however, may be greatly simplified by disengaging it from all accessory elements.

"The location of these hospitals outside of the great centres of population, is the first safeguard, but not an infallible guarantee of their salubrity, while it serves at the same time to preserve each locality from being so many foci of infection." [The author here insists upon the importance of appropriate means of safe and comfortable transportation for the sick.] In France, "the selection of hospital sites is determined by a mixed commission, composed of officers of the line, engineers members of the medical staff, and of the board of health. In electing a site, the following conditions are observed : remoteness from insalubrious spots, elevation of ground, suitable exposure to the sun, proximity to streams of water, vicinity to surrounding groves," &c. It is true, indeed, that we do not always find a combination of all such advantages in hospitals whose ordinary sanitary condition is yet satisfactory; as for example, in certain civil hospitals of London, situated in narrow streets of populous districts; but if an epidemic should arise, the patients would, doubtless, be decimated. It is, therefore, much better to provide, in advance, all the important conditions of salubrity.

The construction of hospitals has a primary importance, regard being had to the choice of the particular spot, to the extent and height of the edifices, and to the arrangement of the several parts of the general plan. But the best contrived construction loses all its advantages as soon as the wards are over-crowded, or even filled with sick, and the extent of the danger necessarily increases with the increasing proportions of the structure. How often has this fact been verified in such large establishments as the Hotel-Dieu, of Paris, and that of Lyons!

The square form of the old military hospitals constructed by Vauban, offering certain advantages in respect to the facilities of supervision and administrative service, is unfavorable for ventilation and salubrity. The tendency at present is to supersede this kind of structure by the more general system of isolated pavilions after the plan of Tenon. The relative separation of the wards prevents the direct propagation of miasmata, and should permit the circumscription, to a certain degree, of the focus of an epidemic. This has been beneficially accomplished at the Val-de-Grace in one of the new pavilions in which, at the date of the latest epidemics, all the cholera patients were collected, and since that period all the sick returning from the Crimea who presented typhoid symptoms.

The height of hospital structures should not exceed two stories, or even one, whenever the price of the ground and the cost of the construction will permit. The temporary army hospitals, made of wood, have but one story near the ground. Such is the construction of the Dey's Hospital, at Algiers, which has been occupied for more than thirty years as a temporary hospital, and its sanitary condition is excellent. The Camp Hospital, at Chalons, and the field hospitals generally, are constructed on this plan, and notwithstanding the necessary lack of some of the important hygienic conditions, the results obtained by means of this simple advantage are truly surprising. The advantages resulting from the superposition of successive stories, have been often insisted upon by Hunter, Coste, Desgenettis, Pastorit and Villirme. It appears, in fact, that a hurtful influence proceeds from the lower to the higher stories. M. Malgaigne has cited a remark of M. Desgenettis, ascribing to the quartering of his patients on the first floor the better success which he had experienced in comparison with a colleague, whose wards were on a higher story. If the wards for the sick ought to be thus separated from each other, it is of equal, or greater importance, that they should be separated from the hospital dependencies, such as the dispensary, the kitchens, the laundry, the bath-house, and the offices generally.

But the proximity of the latrines is inevitable, and often very deleterious. Of all the dependencies of a hospital, this presents the greatest difficulty for its practical adjustment. "In this matter, public hygiene is still behind private hygiene," as has been observed by M. Bouchardat in his lectures on Hygiene. [The author does not suggest any mode of getting rid of the deposits. If a stream of running water can be had, all the difficulties of the problem will be readily solved. Fresh water immediately deodorizes fæcal excrements, and it is only necessary to dam up a portion of the stream immediately above the latrines and to open the flood-gate occasionally, say twice a day, to carry off all adhering masses. In the absence of this inestimable convenience of a running stream, the most effective plan is to have the latrines policed twice a day, and to bury the deposits.]

The staircases and passages of hospitals should be wide and well lighted, in order to facilitate access to the wards at all hours, and to permit free manipulation of the litters.

The arrangement of the wards is one of the most essential points of the question of hospital hygiene. The old system of very capacious wards, admitting many rows of beds pressed closely together, might satisfy spectators, or subserve the convenience of the hospital attendants, but must assuredly compromise the safety of the sick and the responsibility of the surgeons. The appropriation of each building to a limited number of apartments, and above all, the requirement of a maximum limitation of the number of beds for each apartment, cannot be too earnestly recommended to the higher authorities as the best means of ameliorating the most defective hospitals, of perfecting those constructed on the best plan, and of preserving them from the dangers of over-crowding with a certain guaranty of a diminished mortality. The capacity of the apartments might be limited to ten, fifteen, or twenty beds, or might be extended to thirty or forty, but under no circumstances should exceed fifty beds. The separation of such wards by passages, vestibules, or private chambers reserved for cases of peculiar gravity, furnishes conditions favorable to salubrity by establishing the independence of the different services. In all military hospitals, a positive regulation should require the separation of three classes of patients: the sick, or those affected with febrile diseases, and the wounded, and the patients having venereal diseases. A secondary obligation prescribes the isolation of eruptive and contagious diseases, such as small pox. [In this country, where re-vaccination, at stated intervals, is not commonly practised, the obligation to isolate small pox patients is felt to be of primary importance.]

At the present day, hospitals are never built as heretofore, with immense wards communicating directly with each other, thus amalgamating their miasmata at the expense of the sick.

The cubical dimensions of the wards, and the proportion to the bed, constitute a most essential element for the prevention of the evils of over-crowding. The proper allowance should never be diminished under the pretext of accommodating a larger number of sick during the prevalence of epidemics, since this would be equivalent to augmenting the chances of mortality. It is the cubical capacity, and not the superficial area, which ought to determine the number of beds to be placed in each ward. Their cubical capacity should be *at least* thirty metres (*about* 1060 *cubical feet*) to each bed *as a minimum*, and more when ever it can be obtained.—[An interesting and instructive paper on the "site and construction of hospitals" in the British and Foreign Medico-Chirurgical Review, for April, 1860, has this statement: "The cubical space for each patient in this climate has been fixed by European sanitary science at not less than *fifteen hundred feet.*]

"The space between the beds in the same row, should be at least three feet in width, and that between the rows should be from six to nine feet. Ampler dimensions will even be required in the surgical wards.

"The regular and successive evacuation of the wards of a military hospital is of the first importance. It reduces for the time the number of the sick, and furnishes an opportunity for disinfecting and cleansing the wards in succession by white-washing and other suitable means."

The author abstains from any formal notice of the beneficial influences of light, heat and ventilation, on the salubrity of hospitals, the preservation of the lives of the patients, and the curability of their diseases, and of the means of heating and ventilating hospital-wards, in view of the facts that these questions had been fully discussed by M. Bondin in the 47th volume of the "Annals d'Hygiene Publique." He remarks, however, that of all the ingenious novel proposals for improving the ventilation of hospitals, "none have thus far seemed to exert any sensible influence on the diminution of mortality" He concludes, therefore, that natural aëration by means of numerous and opposite windows, as opposed to artificial means of forcing the fresh air into the wards, remains the most simple and the most convenient of all methods yet devised. He enjoins the necessity of moveable openings at the upper part of the windows, which may be obtained by making the upper sash descend by means of cords and pulleys. He recognizes the extreme difficulty in practice of regulating the ventilation in winter, so as to ensure adequate purity of air on the one hand, and protection of the patients from cold draughts on the other. No other duty of the ward-master requires more careful attention and discriminating judgment.

[TO BE CONCLUDED IN NEXT NUMBER.]

